# Bayesian network meta-analysis comparing five contemporary treatment strategies for newly diagnosed acute promyelocytic leukaemia

**DOI:** 10.18632/oncotarget.10118

**Published:** 2016-06-17

**Authors:** Fenfang Wu, Di Wu, Yong Ren, Chongyang Duan, Shangwu Chen, Anlong Xu

**Affiliations:** ^1^ Guangdong Province Key Laboratory for Pharmaceutical Functional Genes, College of Life Sciences, Sun Yat-Sen University, Guangzhou, Guangdong, 510006, People's Republic of China; ^2^ Department of Biostatistics, Southern Medical University, Guangzhou, Guangdong, 510515, People's Republic of China; ^3^ Beijing University of Chinese Medicine, Beijing, 100029, People's Republic of China

**Keywords:** acute promyelocytic leukaemia, Bayesian network meta-analysis, arsenic trioxide, all-trans retinoic acid, chemotherapy

## Abstract

Acute promyelocytic leukemia (APL) is a curable subtype of acute myeloid leukemia. The optimum regimen for newly diagnosed APL remains inconclusive. In this Bayesian network meta-analysis, we compared the effectiveness of five regimens-arsenic trioxide (ATO) + all-trans retinoic acid (ATRA), realgar-indigo naturalis formula (RIF) which contains arsenic tetrasulfide + ATRA, ATRA + anthracycline-based chemotherapy (CT), ATO alone and ATRA alone, based on fourteen randomized controlled trials (RCTs), which included 1407 newly diagnosed APL patients. According to the results, the ranking efficacy of the treatment, including early death and complete remission in the induction stage, was the following: 1. ATO/RIF + ATRA; 2. ATRA + CT; 3. ATO, and 4. ATRA. For long-term benefit, ATO/RIF + ATRA significantly improved overall survival (OS) (hazard ratio = 0.35, 95%CI 0.15–0.82, *p* = 0.02) and event-free survival (EFS) (hazard ratio = 0.32, 95%CI 0.16–0.61, *p* = 0.001) over ATRA + CT regimen for the low-to-intermediate-risk patients. Thus, ATO + ATRA and RIF + ATRA might be considered the optimum treatments for the newly diagnosed APL and should be recommended as the standard care for frontline therapy.

## INTRODUCTION

Acute promyelocytic leukemia (APL) is characterized by a balanced translocation between chromosome 17q21 and chromosome 15q22, leading to an abnormal fusion protein called promyelocytic leukemia-retinoic acid receptor alpha (PML-RARA) [1, 2]. It was not until the introduction of all-trans retinoic acid (ATRA) that most patients achieved complete remission (CR) [1, 3, 4]. ATRA induces the degradation of promyelocytic leukemia-retinoic acid receptor alpha (PML-RARA) oncoprotein. The European APL trial further demonstrated that ATRA plus anthracycline-based chemotherapy (CT) resulted in lower relapse rate [5]. Since then, ATRA+CT treatment has been recommended as the standard care for the newly diagnosed APL [5–11].

Arsenic trioxide (ATO), which acts through specific binding with the PML moiety of the PML-RARA oncoprotein [8, 9], has been shown to be the most effective single agent, and was approved for the treatment of relapsed/refractory APL patients by the Food and Drug Administration (FDA) in 2000. It is not applied in first line therapy because of the limited number of trials and possible side effects reported as hepatic toxicity and prolongation of the QTc interval. However, several recent studies have further tested the efficacy and safety of ATO in newly diagnosed patients, both as single agent and in combination with ATRA. The Italian and German collaboration group (GIMEMA-AMLSG-SAL) has shown that the 2-year overall survival (OS) of ATO+ATRA and ATRA + CT are 99% and 91%, respectively, with no obvious difference in the 2-year event-free survival (EFS). There is also no difference in the incidence of retinoic acid syndromes between the two arms [12]. In a more recent study, ATRA+ATO was compared with ATRA + CT in the randomized National Cancer Research Institute AML17 trial in which a lower dosage of ATO was used, and ATRA + ATO was found to produce a high cure rate with significantly lower liver toxicity [13].

A randomized phase 3 trial performed by the Peking Group found that realgar-indigo naturalis formula (RIF), which contains arsenic tetrasulfide, in combination with ATRA yields similar effectiveness to ATO + ATRA, based on a 3-year OS (99.1% vs. 96.6%) and disease-free survival (DFS) (98.1% vs. 95.5%) [14].

At present, the optimum treatment for APL remains unclear. In this meta-analysis, we pooled and analyzed the comparable RCTs studies, including five contemporary treatments, and aimed to confirm the optimum strategy as the frontline therapy for newly diagnosed APL patients.

## RESULTS

**Figure 1:**
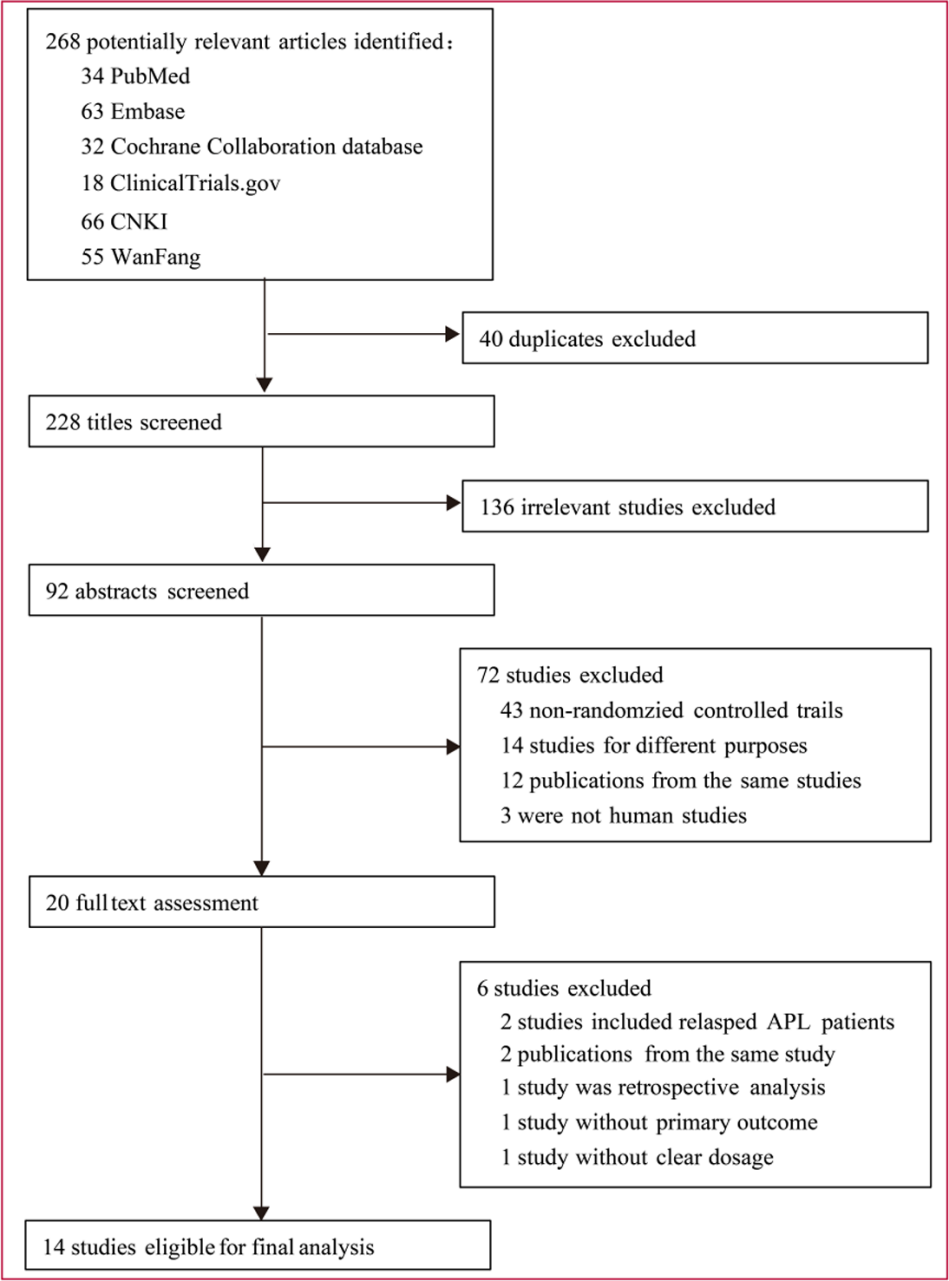
Identification of eligible randomized trials

Fourteen RCTs with 1407 participants were included after the assessment of 268 studies (Figure 1). During the first 3-round selections, 248 studies were excluded. Among these, 40 studies contain duplication, 136 studies are irrelevant, 48 studies are non-RCTs, 14 studies have different purposes, 7 studies have incomplete data on diagnosis or outcomes, and 3 studies are not concerned with human. We then proceeded to check the 20 remaining studies by reading the full texts. Six of these studies were further excluded: two were concerned with relapsed APL patients [15, 16], one with retrospective analysis [17], one without the primary outcome [18], one without clear dosage [19], and one contains duplication [20]. Finally, 14 RCTs with a total of 1407 newly diagnosed APL patients published between 1998 and 2015 were found to be eligible for this meta-analysis [5, 12–14, 21–30]. The patients featured in these studies received different treatments as followed: 537 patients received ATO+ATRA, 117 patients received RIF + ATRA, 297 patients received ATRA + CT, 346 patients received ATRA alone and 110 patients received ATO alone.

Table 1 shows the main characteristics of the randomized trials included in the meta-analysis. Table 2 describes the assessment of the quality of all eligible RCTs. Adequate random sequence generation was found in 9 RCTs (64%). Only 2 RCTs mentioned allocation concealment and 5 RCTs (36%) mentioned intention-to-treat (ITT) principle.

**Table 1:** Main characteristics of the randomized trials included in the meta-analysis

Study	Area	Inclusion period	Size	Male/female	Age (SD/range)		WBC		Induction therapy	
					Arm 1	Arm 2	Arm 1	Arm 2	Arm 1	Arm 2
**ATRA+CT vs ATRA**										
Pierre et al. 1999 [5]	Europe	1993–1996	208	104/104	43(7–63)	45(2–64)	1.4(0.3–4.8)	1.3(0.3–4.7)	ATRA25 mg/m^2^/d, iv 60 mg/m^2^/d DNR for 3 days and 200 mg/m^2^/d Ara-C for 7 days, iv	ATRA:45 mg/m^2^/d, po
**ATO vs ATRA**										
Zhi et al. 2004 [21]	Asia	2001–2003	40	21/19	39.5(15–69)	30.5(14–74)	2.7(0.9–40)	3.0(1.2–49.4)	ATO: 0.16 mg/kg/d, iv	ATRA:25 mg/m^2^/d, po
Su et al. 2006 [22]	Asia	2008–2002	66	31/35	33.3(9–55)	31(4–60)	13.6(± 23.9)	11.9(± 21.5)	ATO: 0.16 mg/kg/d, iv	ATRA:25 mg/m^2^/d, po
Li et al. 2014 [23]	Asia	2008–2013	32	19/13	30.1 (± 4.9)	31.2 (± 5.0)	NA		ATO: 10 mg/d, iv	ATRA:40–90 mg/d, po
Li et al. 2015 [24]	Asia	2000–2013	47	27/20	41(18–74)	38(19–65)	NA		ATO:10 mg/d, iv	ATRA:30–50 mg/m^2^/d, po
**ATO+ATRA vs ATRA**										
Zhi et al. 2004 [21]	Asia	2001–2003	41	24/17	34(14–62)	30.5(14–74)	2.1(0.5–52.6)	3.0(1.2–49.4)	ATRA: 25 mg/m^2^/d, poATO:0.16 mg/kg/d, iv	ATRA:25 mg/m^2^/d, po
Ren et al. 2004 [25]	Asia	1999–2002	95	53/42	34(14–68)	32(14–62)	13.6(± 23.9)	11.9(± 21.5)	ATRA: 25 mg/m^2^/d, poATO: 10 mg/kg/d, iv	ATRA:25 mg/m^2^/d, po
Su et al. 2006 [22]	Asia	1998–2002	70	30/40	37.2(1–66)	31(4–60)	15.7(± 20.6)	11.9(± 21.5)	ATRA: 25 mg/m^2^/d, poATO: 0.16 mg/kg/d, iv	ATRA:25 mg/m^2^/d, po
Wang et al. 2008 [26]	Asia	2003–2007	35	NA	38(3–65)		NA		ATRA:25 mg/m^2^/d, poATO: 10 mg/d, iv	ATRA:25 mg/m^2^/d, po
Liang et al. 2011 [27]	Asia	2003–2010	53	26/27	35.3 (± 14.1)	42.6 (± 15.1)	2.7(1.2–6.5)	1.9(1.0–27.5)	ATRA:25 mg/m^2^/d, poATO:0.16 mg/kg/d, iv	ATRA:25 mg/m^2^/d, po
Xie et al. 2013 [28]	Asia	2006–2012	30	NA	34.5(± 6.3)		22.70(± 1.5)	23.10(± 1.2)	ATRA:40 mg/d, poATO:10 mg/d, iv	ATRA:30–90 mg/d, po
Li et al. 2014 [23]	Asia	2008–2013	32	17/15	30.1 (± 4.9)	31.23(± 5.0)	NA		ATRA:40–90 mg/d, poATO:10 mg/d, iv	ATRA:40–90 mg/d, po
Liu et al. 2014 [29]	Asia	2008–2012	70	42/28	33.5 (± 4.8)	34.4 (± 5.5)	2.87 (± 1.43)		ATRA:25 mg/m^2^/d, poATO:0.16 mg/kg/d, iv	ATRA:25 mg/m^2^/d, po
**ATO + ATRA vs ATRA + CT**										
Lo-Coco et al. 2013 [12]	Europe	2007–2010	156	76/80	44.6(19–70)	46.6(18–70)	1.5(0.3–10)	1.6(0.3–9.6)	ATRA:45 mg/m^2^/d, poATO:0.15 mg/kg/d, iv	ATRA:45 mg/m^2^/d, po+ IDA(12 mg/m^2^/day) on days 2,4,6 and 8, iv
Alan et al. 2015 [13]	Europe	2009–2013	235	120/115	47(16–75)	47(16–77)	3.0(0.4–78.2)	2.2(0.4–100.9)	ATRA:45 mg/m^2^/d, poATO:0.25–0.3 mg/kg/d, iv	ATRA:45 mg/m^2^/d, po+ IDA(12 mg/m^2^/day) on days 2,4,6 and 8, iv
**ATO+ATRA vs ATO**										
Zhi et al. 2004 [21]	Asia	2001–2003	41	21/20	34(14–62)	39.5(15–69)	2.1(0.5–52.6)	2.7(0.9–40)	ATRA:25 mg/m^2^/d, poATO:0.16 mg/d, iv	ATO:0.16 mg/d, iv
Su et al. 2006 [22]	Asia	2998–2002	76	37/39	37.2(1–66)	33.3(9–55)	15.7(± 20.6)	13.6(± 23.9)	ATRA:25 mg/m^2^/d, poATO:0.16 mg/d, iv	ATO:0.16 mg/d, iv
Luo et al. 2012 [30]	Asia	2005–2010	28	17/11	35 ± 9		NA		ATRA:40–60 mg/d, poATO:10mg/d, iv	ATO:10 mg/d, iv
Li et al. 2014 [23]	Asia	2008–2013	32	20/12	30.1 (± 4.85)	41(18–74)	NA		ATRA:40–90 mg/d, poATO: 10 mg/d, iv	ATO:10 mg/d, iv
**RIF + ATRA vs ATO + ATRA**										
Zhu et al. 2013 [14]	Asia	2007–2011	231	126/105	33(15–60)	39(15–60)	2.1 (0.3–50)	2.2 (0.3–50)	ATRA:25 mg/m^2^/d, poRIF: 60 mg/kg, po	ATRA:25 mg/m^2^/d, poATO: 0.16 mg/kg, iv

ATRA = all-trans retinoic acid; CT = anthracycline-based chemotherapy; ATO = Arsenic trioxide; RIF = realgar-indigo naturalis formula; WBC = white blood cell; DNR: daunorubicin; IDA: idarubicin; Ara-C:arabinosyl cytosine.

**Table 2:** Quality assessment for the studies included in the meta-analysis

Study	Randomization process	Estimation of sample size	Allocation concealment	Intention to treat analysis	Dropout	Jadad score
Pierre et al. 1999 [5]	Yes	Yes	No	Yes	Yes	3
Zhi et al. 2004 [21]	Yes	Yes	No	Yes	Yes	3
Ren et al. 2004 [25]	Yes	Yes	No	No	No	2
Su et al. 2006 [22]	Yes	Yes	No	No	No	2
Wang et al. 2008 [26]	Unclear	Yes	No	No	No	1
Liang et al. 2011 [27]	Unclear	Yes	No	No	No	1
Luo et al. 2012 [30]	Unclear	Yes	No	No	No	1
Xie et al. 2013 [28]	Yes	Yes	No	No	No	2
Lo-Coco et al. 2013 [12]	Yes	Yes	Yes	Yes	Yes	3
Zhu et al. 2013 [14]	Yes	Yes	No	Yes	No	3
Li et al. 2014 [23]	Unclear	Yes	No	No	No	1
Liu et al. 2014 [29]	Yes	Yes	No	No	No	2
Li et al. 2015 [24]	Unclear	Yes	No	No	No	1
Alan et al. 2015 [13]	Yes	Yes	Yes	Yes	Yes	3

**Figure 2:**
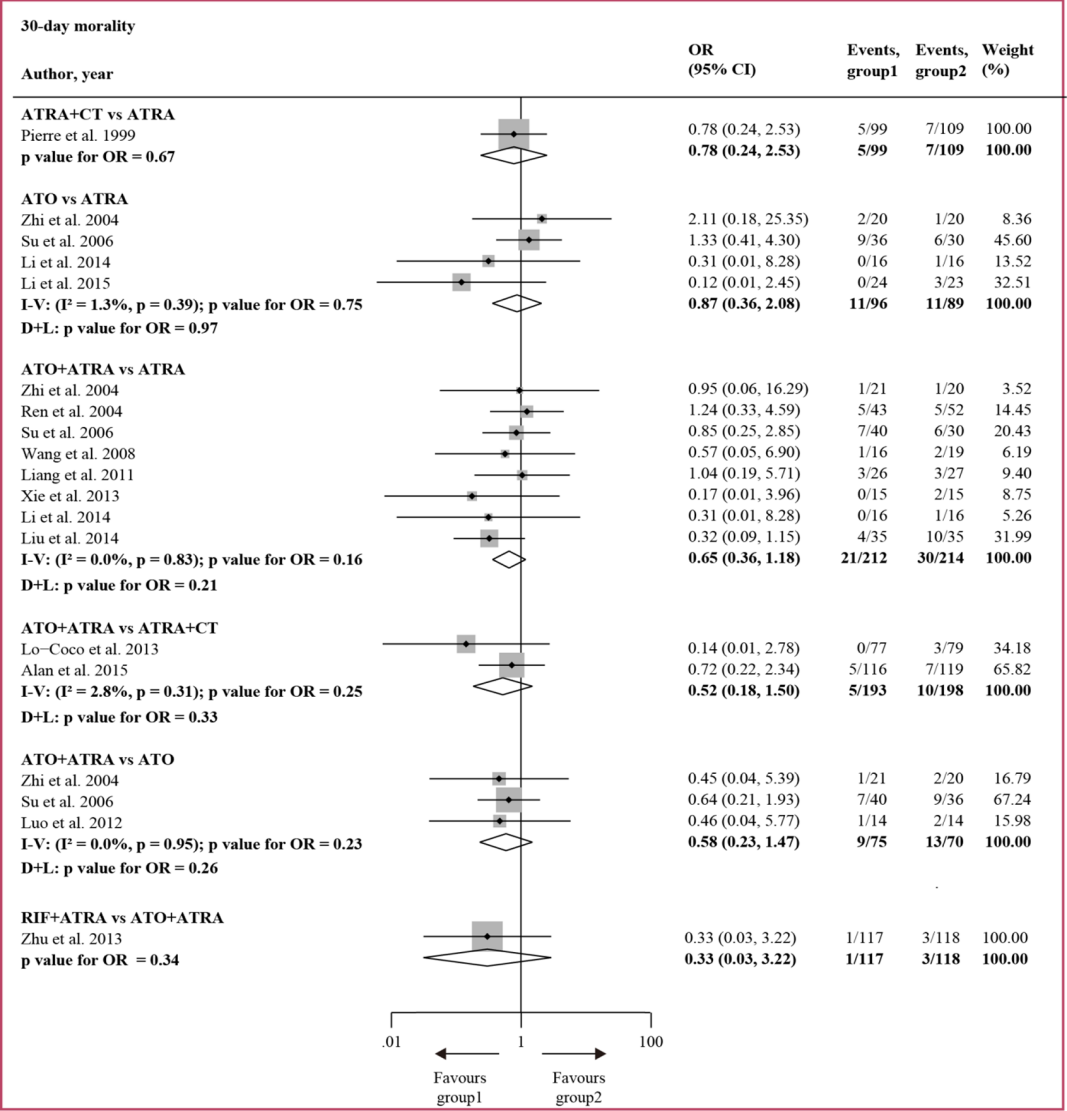
Direct comparisons of treatments based on 30-day mortality I − V = inverse variance. D + L = DerSimonan and Laird. OR = odd ratio.

**Figure 3:**
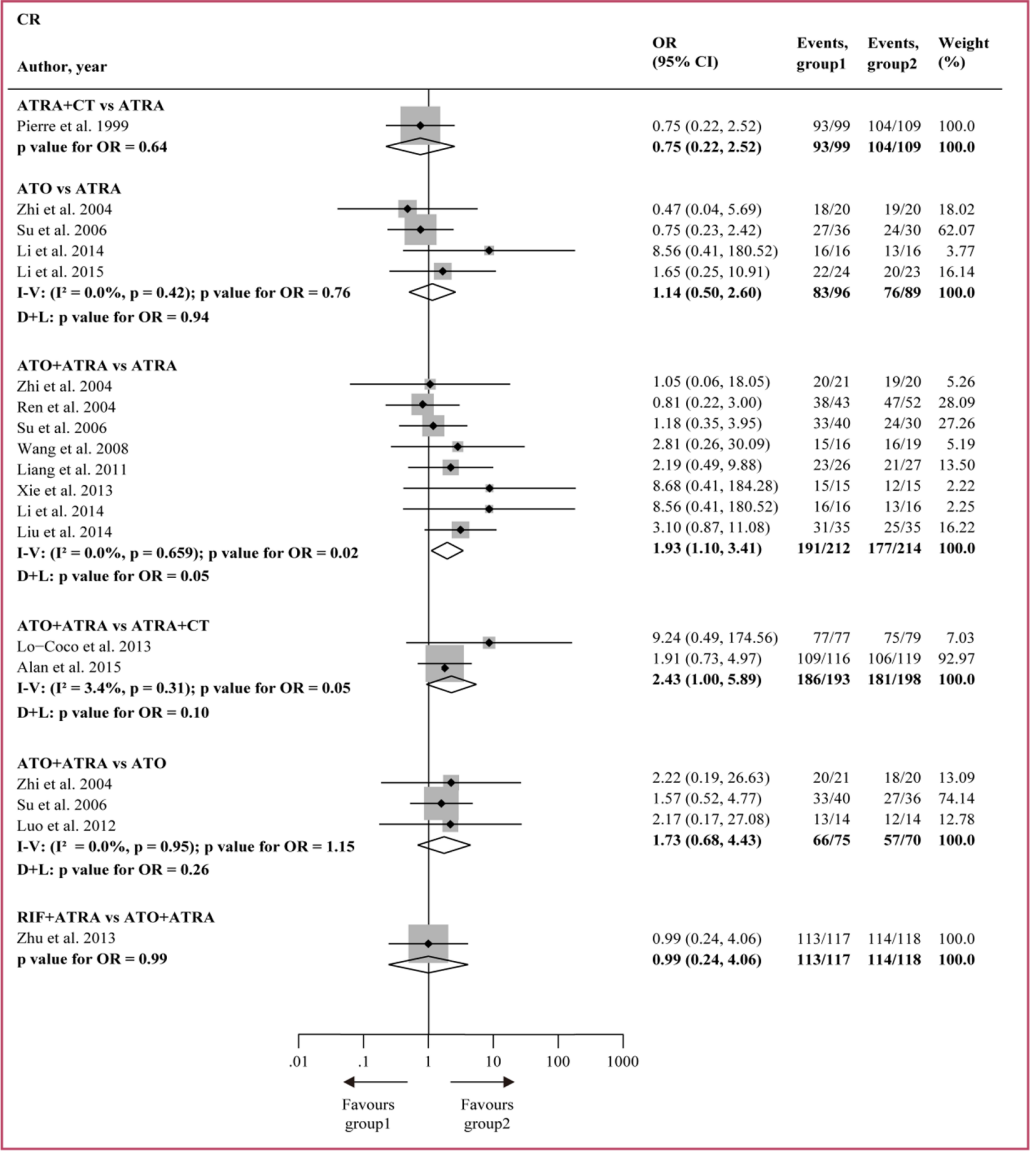
Direct comparisons of treatments based on CR I − V = inverse variance. D + L = DerSimonan and Laird. OR = odd ratio.

Figure 2 and Figure 3 show the direct meta-analysis in the first round of comparison. Of the early deaths reported in the 14 studies, 98 out of 1407 (7.0%) patients died within 30 days, but no significant difference was found in all the pairwise comparisons and no significant heterogeneity was detected. Of the number of CR reported in the 14 studies, 1285 out of 1407 (91.3%) patients achieved CR. ATO + ATRA improved CR compared to ATRA (OR = 1.93, 95%CI 1.10–3.41, *P* = 0.02) and ATRA + CT (OR = 2.43, 95%CI 1.00–5.89, *P* = 0.05), and no significant heterogeneity was found.

**Figure 4:**
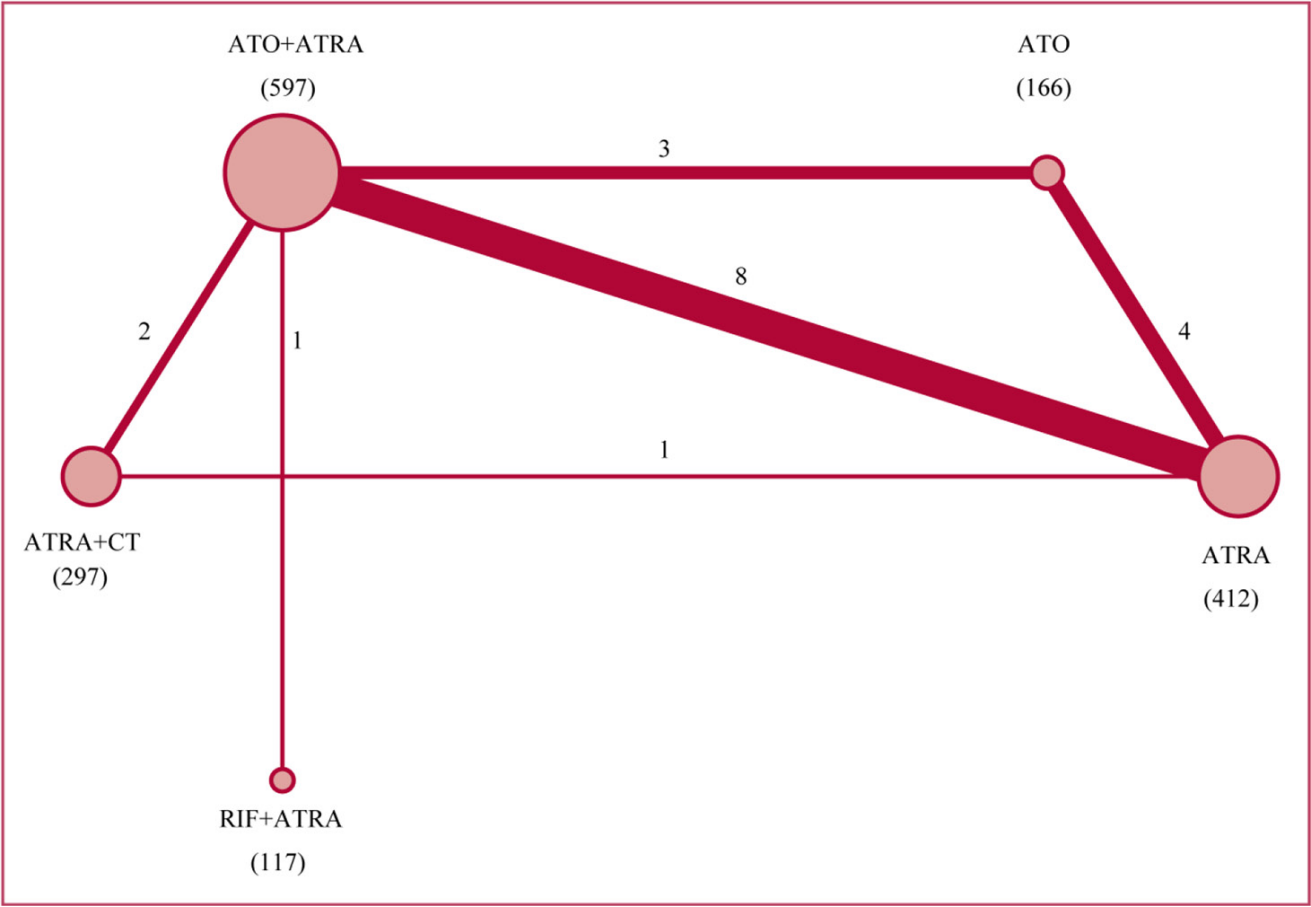
Network of the comparison scheme for Bayesian network meta-analysis The size of the node is proportional to the number of patients randomly chosen for the treatment. The width of the lines is proportional to the number of trials (beside the line) comparing the connected treatments.

**Figure 5:**
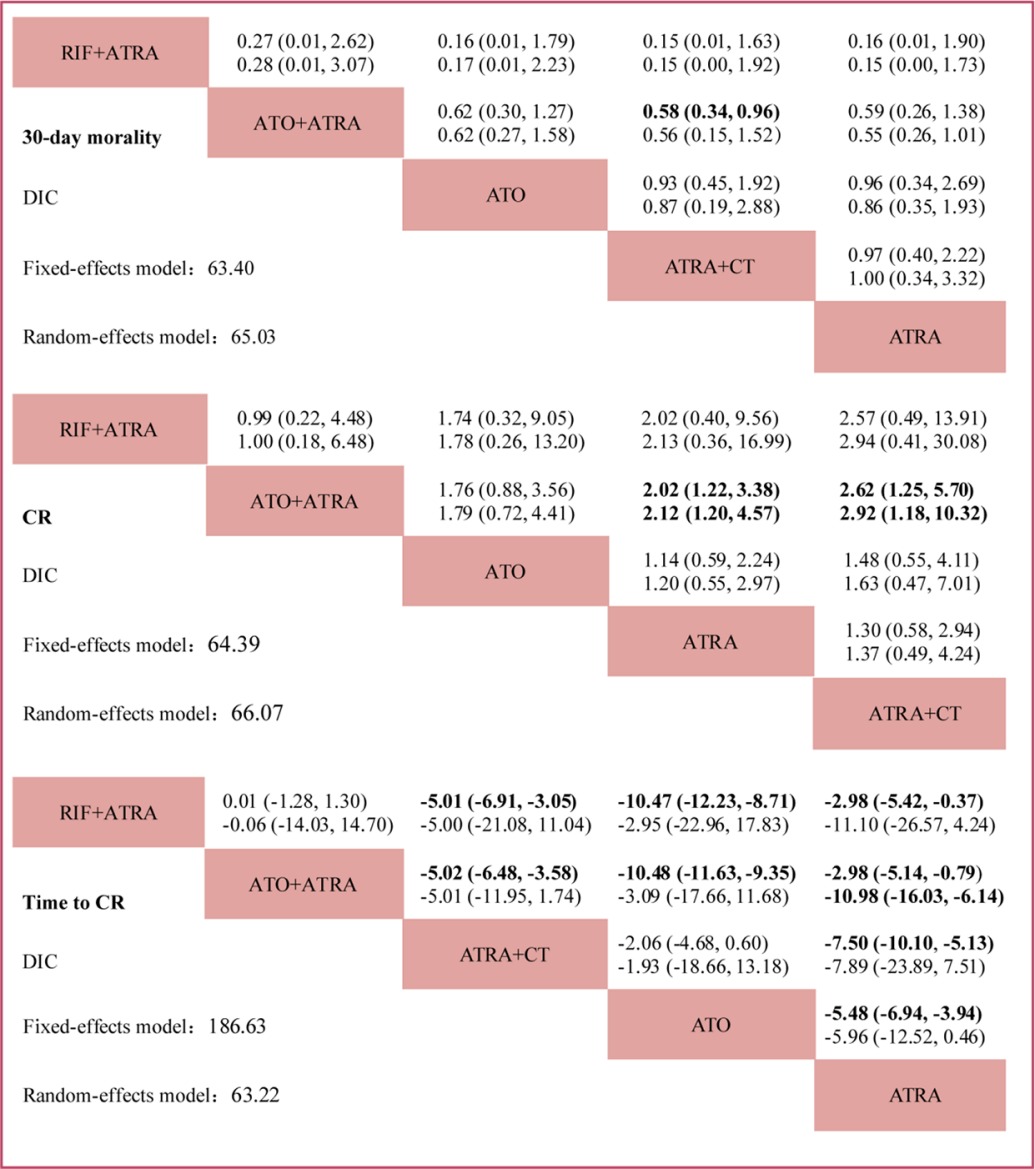
Network of the comparison for 30-day mortality, CR and time to CR The column treatment is compared with the row treatment. In each cell, the first line used fixed-effects model, and the second line used random-effects. Numbers in parentheses indicate 95%CIs. OR/WMD with Bayesian *p* value < 0.05 are in bold.

**Figure 6:**
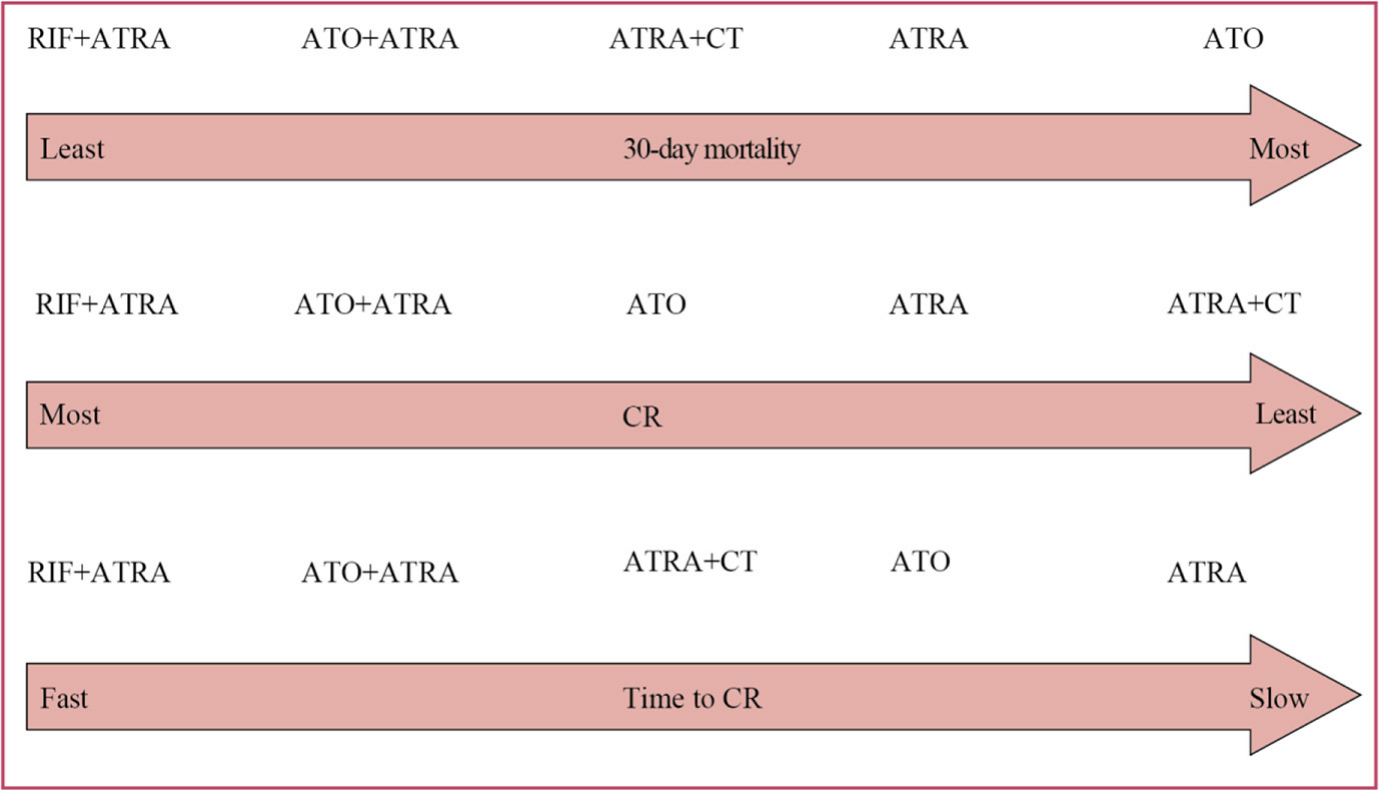
Ranking of treatments in terms of 30-day mortality, CR and time to CR Each treatment was ranked by the percentage of 50,000 iterations.

Figure 4 shows the network of comparison in the Bayesian network meta-analysis. Figure 5 summarizes the results of the network meta-analysis. Both fixed and random effects models were applied. The respective sets of odds ratio (OR) and weighted mean differences (WMDs) with corresponding 95% CrIs from the fixed- and random-effects models showed good consistency despite the relatively wider CrIs of the latter. Moreover, direct and indirect comparisons showed good coherence for all end points, and node-splitting analysis showed no obvious inconsistency (all *P* > 0.05). For early death and CR, the data fitted the fixed-effects model better than the random-effects model according to the DIC (differences were between 1 and 2), with relatively lower values for all end points, indicating that heterogeneity might not be obvious. Furthermore, as both models yielded consistent conclusions, we applied the fixed-effects model for early death and CR, and the random-effects model for time to CR. Figure 6 shows the ranking of each treatment in order of decreasing effectiveness. For early death, RIF + ATRA ranked lowest, followed by ATO + ATRA, ATRA + CT, ATO and ATRA. No significant difference was found among these treatments, except between ATO+ATRA and ATRA + CT. ATO + ATRA reduced the risk of early death compared to ATRA + CT (OR = 0.576, 95% CI = 0.34–0.964 for 30-day mortality). For CR, ATO + ATRA provided an obvious advantage over ATRA alone (OR = 2.023, 95% CI 1.27–3.382) and ATRA + CT (OR = 2.619, 95% CI 1.245–5.7), and the ranking was similar to that of early death. For time to CR, RIF+ATRA and ATO + ATRA retained the advantage. ATRA+CT, however, yielded shorten time to CR than the individual agents. The cumulative probabilities for the most efficacious treatments measured in terms of early death, CR, time to CR during the induction stage were as followed: RIF + ATRA (86%, 49%, 42%), ATO + ATRA (11%, 48%, 32%), ATRA + CT (2%, 0%, 23%), ATO (1%, 3%, 3%), ATRA (0%, 0%, 0%). Node-splitting analysis indicated good coherence and no significant inconsistency between direct and indirect comparisons.

**Figure 7:**
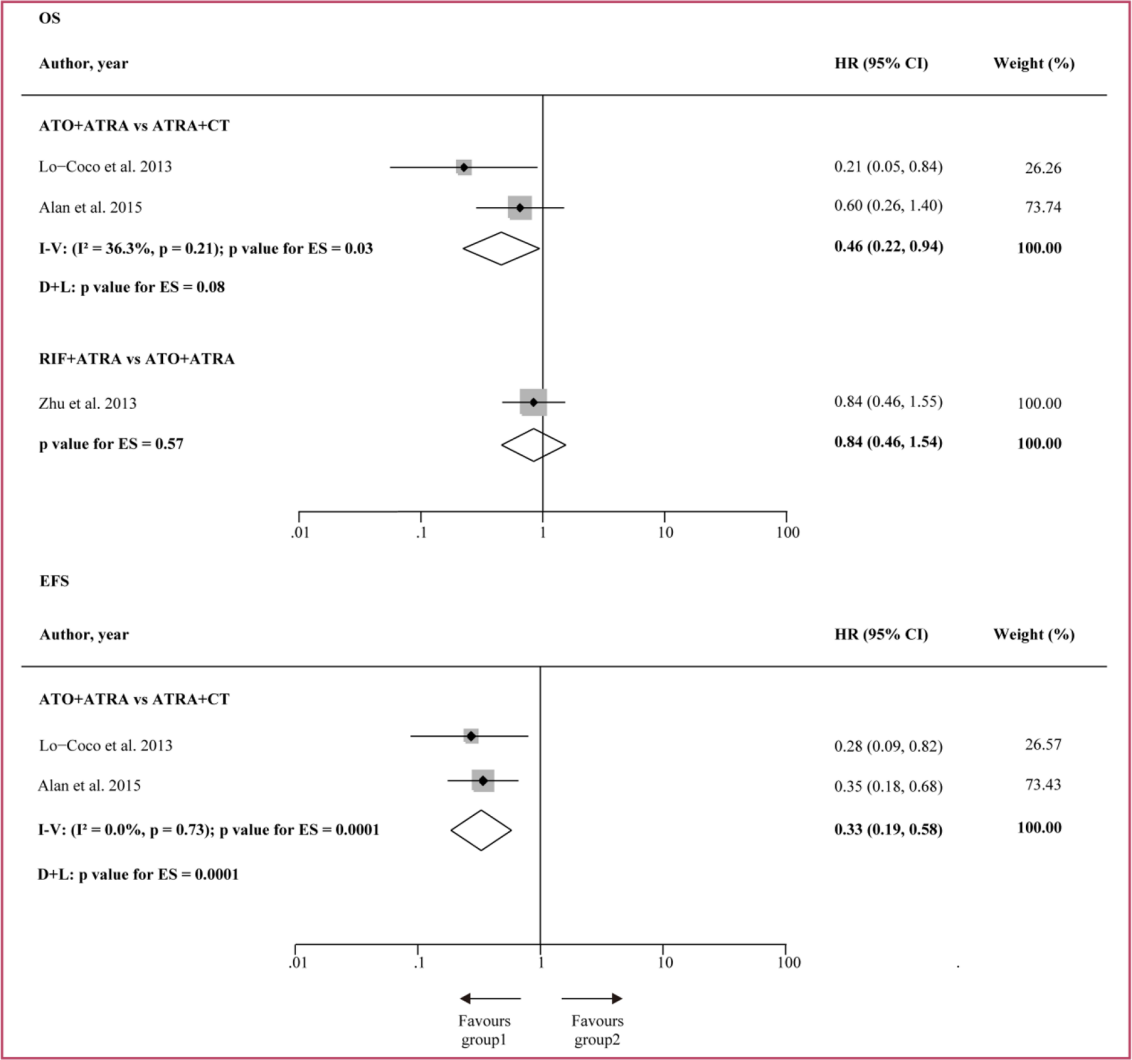
Direct comparison for EFS and OS HR = hazard ratio. I − V = inverse variance. D + L = DerSimonan and Laird. ES = effect estimate for the randomised treatment comparison.

In Figure 7, the OS and EFS of three combination treatments were further compared using direct meta-analysis in the second round of comparison. For OS, HR was reported by AML17 and could be estimated in the other two studies. ATO + ATRA significantly improved OS over ATRA + CT (HR = 0.46, 95% CI 0.22–0.94, *p* = 0.03) in the fixed effects model. However, moderate heterogeneity was found (I^2^ = 36.3%, *p* = 0.21), probably due to the different risk levels of patients and the different dosages used in the two studies. The patients in APL0406 studies were all low-to-intermediate-risk APL, while 24% of the patients included in AML17 were high-risk APL. In the comparison between RIF + ATRA and ATO + ATRA, no significant difference in OS was found. For EFS, HR was explicitly reported by AML17 and could be estimated in the APL0406 study. ATO + ATRA was found to have an advantage in EFS over ATRA + CT (HR = 0.33, 95%CI 0.19–0.58, *p* = 0.001).

**Figure 8:**
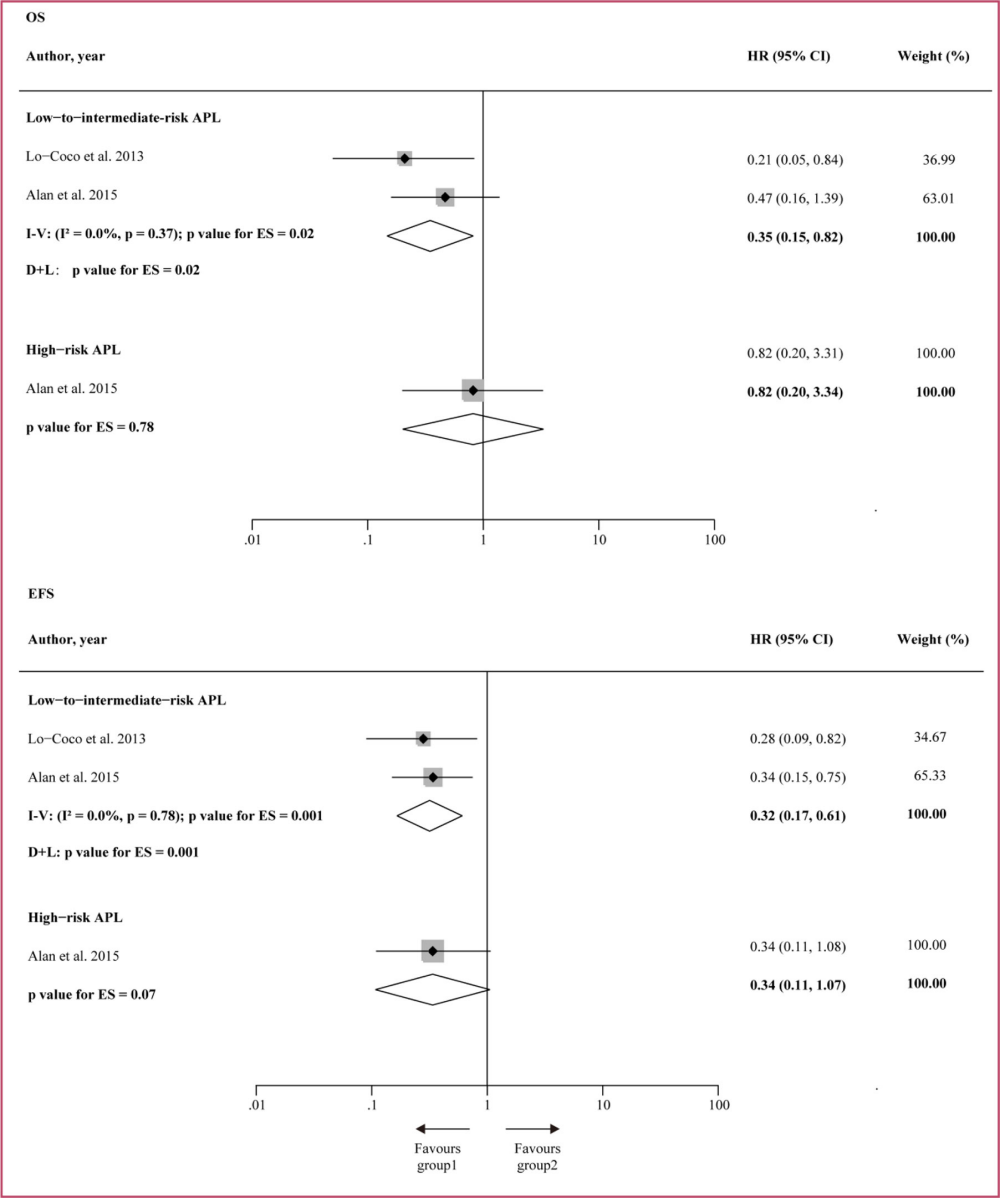
Subgroup analysis for the two combination treatments on the low-to-intermediate-risk (WBC ≤ 10 × 10^9^/L) and high-risk (WBC > 10 × 10^9^/L) patients HR = hazard ratio. I − V = inverse variance. D + L = DerSimonan and Laird.

Given to different risk levels of APL patients in the two studies, subgroup analysis for the two combination of treatments was further conducted for the low-to-intermediate-risk patients (WBC ≤ 10 × 10^9^/L) as well as for the high-risk patients (WBC > 10 × 10^9^/L), and the result is presented in Figure 8. For the low-to-intermediate-risk APL patients, ATO + ATRA significantly improved both OS (HR = 0.35, 95%CI 0.15–0.82, *p* = 0.02) and EFS (HR = 0.32, 95%CI 0.16–0.61, *p* = 0.001) over ATRA + CT. For the high-risk APL, however, no significant difference was found between ATO + ATRA and ATRA + CT.

## DISCUSSION

The relevant meta-analysis of treatment strategies for newly diagnosed APL published in recent years mostly compared ATO+ATRA with the single agent (ATO or ATRA), but not with the standard treatment (ATRA + CT) [31–33]. In contrast, our meta-analysis assessed, for the first time, five contemporary treatments for newly diagnosed APL. It showed that three combinations of treatment strategies (RIF + ATRA, ATO + ATRA and ATRA + CT) were superior to the two single-agent treatments, ATO or ATRA alone in the induction phase [34–36]. OS and EFS were assessed to determine the long-term efficacy of the treatments. ATO + ATRA significantly improved OS and EFS over ATRA + CT, but this was only observed for the low-to-intermediate-risk patients, and not for the high risk patients. In addition, both ATO + ATRA and RIF + ATRA tended to be optimum with respect to the best survival rate for the induction phase therapy and long-term efficacy and safety.

Due to the possible side effects of hepatic toxicity and prolonged QTc interval, ATO was approved only for the refractory or relapsed APL patients in the US and Europe. However, the AML17 trial has shown that an attenuated dosage of ATO performed during the induction and consolidation phases can obviously reduce the risk of ATO-based adverse effects [13]. ATO + ATRA yields less serious adverse effects than ATRA + CT [13]. These results show that for long-term safety with minimized toxicity, ATO + ATRA is also superior to ATRA+CT.

Health-related quality-of-life (HRQOL) can help physicians make more informed therapy decisions for APL patients. Lo-Coco stated in his recent meta-analysis of randomized clinical trials in APL that the benefits of HRQOL provided by ATO + ATRA over standard ATRA + CT are mainly valid at the end of the induction phase [38] However, HRQOL assessment is highly challenging because of the large amount of missing data. Long-term HRQOL might be better for patients treated with ATO + ATRA than with ATRA + CT because the former means that patients do not need to receive maintenance therapy for the following successive 2 years. Thus study on HRQOL is urgently needed to further investigate the benefit of APL treatments.

A recent Bayesian network meta-analysis that evaluated the efficacy of different APL treatments has suggested that ATO- or RIF-based treatment strategy may be the best therapy [38]. Our meta-analysis differs from that study in several ways. Firstly, for the long-term efficacy in our study, we used hazard ratio (HR) with the 95% CI, which is the only summary statistics that allows for both censoring and time to an event. Secondly, we included an important trial—AML17, which could explain the different incidences of adverse side effects induced by ATO-based treatment. Finally, the limitation of this work should be noted. All the data we collected were from RCTs published online instead of individual patient data, and this may result in publication or report bias. In addition, even though we have pooled all the relevant RCT studies, sampling bias could not be avoided because of the limited data, suggesting further trials are needed to draw a clear cut conclusion.

## MATERIALS AND METHODS

### Search strategy and selection criteria

We searched PubMed, Cochrane database, Embase, ClinicalTrials.gov, Google Scholar until the end of December 2015 without language restrictions. In addition, we also searched Chinese periodicals, including China National Knowledge Infrastructure (CNKI) and WanFang database. The terms used in the search subjects were as follow: “randomized clinical trial”, “APL” or “acute promyelocytic leukemia” or “M3”, “ATO” or “arsenic trioxide” or “arsenic” or “Trisenox”, “Realgar-Indigo naturalis formula” or “RIF” or “arsenic tetrasulfide”, “ATRA” or “all-trans retinoic acid”, “chemotherapy” or “CT”.

In our meta-analysis we included the trials that compared two or more of the five treatments for the newly diagnosed APL. Non-randomized trials or studies that contain only one or none of the five strategies were excluded.

### Data extraction and quality assessment

Two investigators (F.W. and D.W.) independently reviewed and checked the included studies to ensure the quality of the data. We extracted the relevant data of eligible RCT studies into an electronic database. The extracted information included patient details, inclusion and exclusion criteria, treatment protocols, and outcomes (early death, complete remission rate, the time to achieve complete remission for the first round comparison and OS, EFS for the second round). For early death and CR, the number of dead patients within 30 days and the number of patients assessed in each treatment group were recorded. For time to achieve CR, the arithmetic means and standard deviations (SDs) were extracted for each treatment group, together with the number of patients assessed in each group. For OS and EFS, extraction of summary statistics was performed according to the methods described by Parmar et al. [39].

To assess the quality of RCTs, the following five components were examined: randomization procedure; estimation of sample size; allocation concealment; incomplete outcome data; and whether the intention-to-treat analysis was being followed; and loss to follow-up and dropout. Jadad/Oxford quality scoring system was used to quantify the study quality [40]. Disagreement was resolved by all participants until a consensus was reached.

### Statistical analysis

Both direct pairwise meta-analysis and network meta-analysis were performed to evaluate the outcomes. Comparable RCTs performed with similar induction regimens were grouped for first-round meta-analysis. Based on the result of the first-round comparisons, the comparable RCTs performed with similar induction and consolidation regimens were further grouped for the second round of comparison.

The treatment effect for early death and complete remission were analyzed by odds ratio (OR) with 95% confidence intervals (CI), and the time to achieve CR were analyzed by weighted mean differences (WMD) with 95% CIs. OS and EFS were analyzed by HRs with 95% CIs, with time-to-event information and confounders being adjusted for. The weight given to each study was determined by the precision of its estimate of effect and was equal to the inverse of the variance. A two-sided *P*-value of < 0.05 was considered significant. Statistical homogeneity of effects across studies was assessed using the Cochran Q statistic and I^2^ statistic along with forest plot. I^2^ with suggested thresholds for low (0–25%), moderate (25–50%), and high (≥ 50%) heterogeneity. The pooled effects were calculated with both fixed effect (inverse variance weighted) and random effect (DerSimonian and Laird) models. Direct meta-analysis was conducted by using Review Manager Version 5.3 (Revman; the Cochrane Collaboration; Oxford, England).

For network meta-analysis, the treatment effects were estimated by posterior means with corresponding 95% credible intervals (CrIs), which can be interpreted as conventional 95% confidence intervals (CIs) [41]. Both fixed and random effects models were used, and then assessed by the Bayesian deviance information criterion (DIC) statistics [42]. Non-informative uniform and normal prior distributions were used to fit the data to the models, yielding 50,000 iterations with a burn-in number of 10,000 iterations and a thin interval of 50 to obtain the posterior distributions of the model parameters. Convergence of iterations was assessed with the Gelman-Rubin-Brooks statistic [43]. The probability of each treatment in the ranking was estimated based on its posterior probabilities, which depended on counting the proportion of iterations in the Markov chain of OR ranking in the treatments. To assess whether there was inconsistency between direct and indirect comparisons, the pooled ORs from the network meta-analysis were compared with the corresponding ORs from traditional pair-wise random-effects meta-analysis of direct comparisons. Node-splitting analysis was also applied to evaluate the inconsistency for closed loops in the network [44, 45]. Significant inconsistency between direct and indirect evidence was indicated by node-splitting analysis (*P* < 0.05). The network meta-analysis were built in WinBUGS 1.4.3 (MRC Biostatistics Unit, Cambridge, UK) [46].
